# Unfermented Freeze-Dried Leaf Extract of *Tongkat Ali* (*Eurycoma longifolia* Jack.) Induced Cytotoxicity and Apoptosis in MDA-MB-231 and MCF-7 Breast Cancer Cell Lines

**DOI:** 10.1155/2021/8811236

**Published:** 2021-01-30

**Authors:** Lusia Barek Moses, Mohd Fadzelly Abu Bakar, Hasmadi Mamat, Zaleha Abdul Aziz

**Affiliations:** ^1^Faculty of Applied Sciences and Technology, Universiti Tun Hussein Onn Malaysia (UTHM), Pagoh Campus, Hub Pendidikan Tinggi Pagoh, KM1, Jalan Panchor, 84600, Muar, Johor, Malaysia; ^2^Institute for Tropical Biology and Conservation, Universiti Malaysia Sabah, Jalan UMS, Kota Kinabalu, Sabah 88400, Malaysia; ^3^Faculty of Food Science and Nutrition, Universiti Malaysia Sabah, Jalan UMS, Kota Kinabalu, Sabah 88400, Malaysia; ^4^Faculty of Science and Natural Resources, Universiti Malaysia Sabah, Jalan UMS, Kota Kinabalu, Sabah 88400, Malaysia

## Abstract

The present study was conducted to determine the cytotoxicity effect of *Eurycoma longifolia* (Jack.) leaf extracts and also its possible anticancer mechanism of action against breast cancer cell lines: non-hormone-dependent MDA-MB-231 and hormone-dependent MCF-7. The leaves of *E. longifolia* were processed into unfermented and fermented batches before drying using freeze and microwave-oven drying techniques. Obtained extracts were tested for cytotoxicity effect using MTT assay and phenolic determination using HPLC-DAD technique. The most toxic sample was analyzed for its apoptotic cell quantification, cell cycle distribution, and the expression of caspases and apoptotic protein using flow cytometry technique. Fragmentation of DNA was tested using an agarose gel electrophoresis system. The results determined that the unfermented freeze-dried leaf extract was the most toxic towards MDA-MB-231 and MCF-7 cells, in a dose-dependent manner. This extract contains the highest phenolics of gallic acid, chlorogenic acid, ECG, and EGCG. The DNA fragmentation was observed in both cell lines, where cell cycle was arrested at the *G*_2_/M phase in MCF-7 cells and S phase in MDA-MB-231 cells. The number of apoptotic cells for MDA-MB-231 was increased when the treatment was prolonged from 24 h to 48 h but slightly decreased at 72 h, whereas apoptosis in MCF-7 cells occurred in a time-dependent manner. There were significant activities of cytochrome c, caspase-3, Bax, and Bcl-2 apoptotic protein in MDA-MB-231 cells, whereas MCF-7 cells showed significant activities for caspase-8, cytochrome c, Bax, p53, and Bcl-2 apoptotic protein. These results indicate the ability of unfermented freeze-dried leaf extract of *E. longifolia* to induce apoptosis cell death on MDA-MB-231 and MCF-7, as well as real evidence on sample preparation effect towards its cytotoxicity level.

## 1. Introduction

Globally, 1 in 6 deaths is due to cancer, which ranked it as the second leading cause of death [1]. According to GLOBOCAN produced by the International Agency for Research on Cancer, out of 185 countries, approximately 18.1 million new cancer incidence and 9.6 million deaths were reported in 2018 [2]. The most commonly diagnosed is lung cancer, with 2.09 million cases and closely followed by female breast cancer, also with 2.09 million cases. Breast cancer is ranked fifth for mortality with 627 000 deaths after lung cancer (1.76 million deaths), colorectal cancer (862 000 deaths), stomach cancer (783 000 deaths), and liver cancer (782 000 deaths) [1]. By 2040, the cancer incidence expected to grow to 27.5 million new cases and 16.3 million deaths due to the growth and ageing of the population [3]. As the second leading of diagnosed cancer, breast cancer has increased concern worldwide, especially among females. However, the frequency of its diagnosed and mortality cases significantly varied across countries and within each country, depending on the degree of economic development as well as the social and lifestyle factors. The reported incidences have been occurring in both developed and less developed countries, where almost 70% of deaths occur in less developed countries [1]. The GLOBACAN of the International Agency for Research on Cancer has estimated the age-standardized rate (ASR) of breast cancer in Malaysia as 38.7 per 100,000 with 5,410 new cases in 2012 [4].

Traditional and complementary medicine has also been suggested as an alternative treatment besides surgery, chemotherapy, and pharmacogenomics therapy to reduce the breast cancer occurrence [5]. Up to 64% of traditional and complementary medicines, uptakes were reported by women with breast cancer. However, up to date, only a few traditional or complementary therapies have been tested scientifically [4]. In the early stage of developing an anticancer drug, a study on the biochemical reaction of a sample and its mechanism of action is crucial, especially on the determination of its cell death mode [6]. Apoptosis is a natural programmed cell death mode triggered by anticancer drugs as well as other physical and chemical factors [7]. Once apoptosis showed a defective regulation, it would lead to an uncontrollable proliferation of cancer cell [8, 9]. Therefore, regulated apoptosis becomes a major target and principal mechanism in the development of an effective anticancer chemotherapeutic agent [6].

*Eurycoma longifolia* Jack. (Simaroubaceae family) is prevalent among traditional medicinal practitioners. This medicinal plant is commonly known as Tongkat Ali (Malaysia), Pasak Bumi (Indonesia), Tung Saw (Thailand), Cay Ba Binh (Vietnam), Tho Nan (Laos), and Babi Kurus (Java) [10]. The decoction of this plant is mainly used to increase energy and vitality for man and as a tonic for a woman after childbirth. Some of its uses are to treat fatigue, malaria, diarrhoea, dysentery, glandular swelling, bleeding, dropsy, cough, fever, ulcer, and high blood pressure [11, 12]. Scientifically, there have been numerous studies and a wide range of pharmaceutical properties discovered from its roots [13]. It has shown anticancer activities on various types of cancer, including lung, breast, and cervical cancers. Salahi et al. [14] have reported the antitumour activity of *E. longifolia* root extracts against leukemic cell line of K-562. Meanwhile, its branch extract-mediated silver nanoparticles exhibited significant anticancer activity against human glioma cells (DBTRG and U87) and human breast cancer cells (MCF-7 and MDA-MB-231) [15]. Yet, the utilization of its leaf remains minimal as it usually discarded after harvesting its root. Hence, utilizing its leaf by preparing it as herbal tea in this present study was to highlight its significance as one of the potential alternative medicines for breast cancer, as well as to maximize the utilization of this plant.

In the meantime, sample preparation also plays a crucial factor to maximize its potential. The fermentation process can cause alteration or reduction of phytochemical content which may affect the anticancer potential. Apart from fermentation, drying technique also plays an essential factor in the quality preservation of the end-product. Efficient drying techniques will enhance the quality of dried product such as aroma and appearance by hindering any biochemical changes or microbial growth [16]. Through this study, the most effective procedure of processing and preparation of *E. longifolia* leaves to obtain the optimum cytotoxicity effect against cancer cells were outlined.

## 2. Materials and Methods

### 2.1. Preparation of Unfermented and Fermented Leaves

About 1.0 kg of fresh intermediate leaves (from 2^nd^ axis to 4^th^ axis) of *Eurycoma longifolia* leaves was collected from the hilly area of Universiti Malaysia Sabah (UMS), and a voucher specimen was deposited into BORNEENSIS Herbarium (BORH), Institute for Tropical Biology and Conservation in UMS. The leaves were washed with distilled water and blotted with a paper towel to remove excess water. Then, the cleaned leaves were divided into two batches (approximately about 0.5 kg/batch) of unfermented and fermented leaves. The unfermented and fermented leaves were prepared as described by Lusia Barek et al. [17]. For unfermented one, the leaves were firstly steam blanched at 98 ± 2°C for 30 sec to inactivate degradative enzymes and immediately soaked in an ice-cold water bath for 30 sec before blended for 5 sec using a blender (Panasonic Mx-337, Malaysia) and dried. Meanwhile, those fermented leaves were firstly left in the open air for 18 hours before ground using a blender for 5 sec. The ground leaves were then sprayed with distilled water in a ratio of 1 : 1 (w/v) and left in dark condition at room temperature (25 ± 1°C) for 5 h to undergone oxidation-fermentation process.

For drying, each unfermented and fermented leaves of *E. longifolia* were further divided into two batches and dried using microwave-oven and freeze-drying techniques. For microwave-oven dried, ground leaves were dried using a microwave-oven (Samsung MW71 E, South Korea) at 600 W for 5 min; meanwhile, freeze-dried leaves were firstly frozen at −80°C for 48 h before subjecting into the freeze dryer (Labconco FreeZone, United State) for 48 h.

The leaf extract was prepared by infusing 2.0 g of dried leaves in 200 ml boiling distilled water (100 ± 2°C), stirred using a magnetic stirrer (Stuart SD162, United Kingdom) at 300 rpm for 2 min and left to cool for 10 min before filtering through Whatman No.4 filter paper. Resultant infusions were lyophilized by freezing those at −80°C for 48 h before subjecting into the freeze dryer for 48 h. Two commercial tea *Camellia sinensis* products, namely, “BOH green tea” (unfermented) and “SABAH black tea” (fermented), were used as a comparison to *E. longifolia* herbal teas. These teas were prepared as the same as the *E. longifolia* herbal extracts. Each 1.0 g of extracts was dissolved in 1.0 ml dimethyl sulfoxide and stored in −20°C for further used.

### 2.2. Cell Culture

Human breast cancer cells (MCF-7 and MDA-MB-231) and normal mouse embryonic fibroblast, 3T3-NIH, were obtained from the American Type Culture Collection (ATCC) (Manassas, United States). The MCF-7 and MDA-MB-231 cancer cells were maintained in an RPMI 1640 medium supplemented with heat-inactivated fetal bovine serum (FBS) and penicillin-streptomycin in a ratio of 100 : 10 : 1 (v/v/v); meanwhile, the 3T3-NIH normal cell was maintained in DMEM and supplemented as the same as the cancer cells. These cells were kept until they grew exponentially in a humidified cell incubator (Sanyo, Japan) with an atmosphere of 5% CO_2_ at 37°C.

### 2.3. MTT Cytotoxicity Assay

MCF-7, MDA-MB-231, and 3T3-NIH cells were seeded at a concentration of 1 × 10^3^ cells/ml in a 96-well plate and incubated for 24 h in an incubator at 37°C with 5% CO_2_. After incubation, the old medium was replaced with the extract diluted with the respective complete growth medium (200, 100, 50, 25, 12.5, 6.5, and 0.0 µg/ml) and followed by incubation for 72 h. After the incubation, each well was added with 20 *µ*l of 5 mg/ml MTT solution and incubated in dark condition for another 3 h [18]. Then, the medium with the MTT solution was removed, and 100 *µ*l of absolute dimethyl sulfoxide (DMSO) was added to each well to dissolve formazan crystal that has been formed by viable cells. Each well was measured at 540 nm using an ELISA plate reader (FLUOstar Omega, BMG LABTECH, Germany). The percentage of cell viability was calculated as follows:

(1) $$\begin{array}{c} \text{percentage of cell viability}\left(\%\right)=\frac{\text{OD sample}}{\text{OD control}}\times 100\%, \end{array}$$

where OD = optical density, control = without treatment of extract, and sample = with treatment of extract. The IC_50_ values were determined as the concentration of the extracts that caused 50% inhibition or cell death.

### 2.4. HPLC-DAD Analysis for Phenolic Determination

HPLC analysis was performed to determine phenolic content using an HPLC 1200 series equipped with column Agilent ZORBAX Eclipse (XDB-C18), 5 *µ*m, (250 × 4.6 mm internal diameter), G1322 vacuum degasser, G1311 quaternary pump, G1329 autosampler, G1316 thermostatted column compartment, and G1315 diode array detector (DAD) with ChemStation Software. Twelve (12) phenolic standards of *p*-coumaric acid, chlorogenic acid, vanillic acid, ferulic acid, gallic acid, caffeic acid, (-)-epicatechin (EC), (-)-epigallocatechin (EGC), (-)-epicatechin gallate (ECG), (-)-epigallocatechin gallate (EGCG), hesperidin, and naringin were prepared in a series of concentrations ranged from 20 to 100 ug/ml for calibration of individual phenolic quantification. Each *E. longifolia* infusion was filtered through a 0.5 *µ*m nylon membrane filter before injection into the HPLC apparatus. The sample flow rate was set up for 1 ml/min with 20 *µ*l of injection volume. The phenolic content was measured at 280 nm. The mobile phases were composed of (*A*) 10% acetic acid in acetonitrile and (*B*) water, filtered under vacuum through a 4.5 *µ*m membrane filter before used. Gradient elution was performed as 10% solvent A constantly for 9 min before increased to 20% for 2 min. At 12 min, the elution increased to 35% for 4 min before drastically decreased to 10% at 18 min and remained constant for 2 min.

### 2.5. DNA Fragmentation Analysis

Cancer cells at the density of 5 × 10^5^ cells/well were seeded in a 6-well plate and grown for 24 h in an incubator at 37°C with 5% CO_2_. The cells were treated with selected *E. longifolia* leaf extract at IC_50_ value and incubated again for 72 h. The treated cells were harvested and transferred into a Falcon tube before centrifuged at 2000 rpm for 5 min to obtain a cell pellet. The pellet was washed twice with 1x PBS. Positive control of drug doxorubicin was treated similarly as the extract. DNA laddering analysis was performed according to the procedure described in Cayman DNA Laddering Assay Kit (Ann Arbor, Michigan) to obtain a dry cell pellet. Before electrophoresis, the dry pellet was resuspended in 25 *µ*l of TE buffer. Each 10 *µ*l of cell extract was mixed with 2 *µ*l of loading buffer. DNA fragments were separated in 0.8% agarose gel stained with 5.0 mg/ml ethidium bromide at 75 V for 60 min using an agarose gel electrophoresis system. *A* 1 kb plus of DNA ladder (Invitrogen, USA) was used as a marker to determine the DNA fragment size. DNA fragmentation was visualized using under UV light using FluorChem 5500 Chemiluminescent (Alpha Innotech, United State).

### 2.6. Cell Cycle Determination

The cell cycle distributions of treated cells were determined according to Abu Bakar et al. [19]. The cells at the density of 5 × 10^5^ cells/well were seeded in the 6-well plates and incubated at 37°C with 5% CO_2_ for 24 h before treated with selected leaf extract at its IC_50_ values and incubated again for 24 h, 48 h, and 72 h. The treated cells were then harvested after reaching the respective treatment period, washed twice with 1x cold PBS, and centrifuged. Obtained cell pellets were added with 500 *µ*l 1x cold PBS and 4.5 ml of ice-cold 70% ethanol before incubated for overnight at −20°C. The cell pellets were rewashed twice with 1x cold PBS before resuspended in 500 *µ*l propidium iodide staining solution (containing 100 *µ*g/ml propidium iodide and 40 *µ*g/ml RNase). Finally, the cells were incubated for 30 min at 37°C in dark condition before analyzing using Summit 4.3 software of flow cytometer (Alpha Innotech, California, USA).

### 2.7. FITC Annexin V Apoptosis Analysis

The FITC Annexin V apoptosis staining was conducted using FITC Annexin V Apoptosis detection kit (BD Pharmingen, Bioscience, Austria). The cells (5 × 10^5^ cells/well) were seeded in the 6-well plates and grown for 24 h at 37°C with 5% CO_2_ in an incubator. After incubation, the cells were treated with selected leaf extract at IC_50_ values for 24 h, 48 h, and 72 h. All adhering and floating cells were collected into centrifuged tubes before washing twice with 1x cold PBS. The cell pellets were resuspended in 1 ml 1x binding buffer (containing 0.1 M HEPES/NaOH (pH 7.4), 1.4 M NaCl, and 25 mM CaCl_2_). Then, 500 µl was transferred into Cryovial tubes and added with 5 *µ*l of FITC Annexin V and 10 µl of propidium iodide. The tubes were incubated in dark condition at room temperature (25 ± 2°C) for 15 min before analyzing using Summit 4.3 software of flow cytometer.

### 2.8. Caspase-3 and -8 and Cytochrome c Determination Assay

Cells at the density of 5 × 10^5^ cells/well were seeded in the 6-well plates, grown, and treated with selected leaf extract at its IC_50_ values before harvested. The activity of caspase-3 of the tested herbal extract was determined using the caspase-3 assay kit, colourimetric (SIGMA, Germany). Briefly, the harvested cells were then lysed using 1x lysis buffer (containing 250 mM HEPES, pH 7.4, 25 mM CHAPS, and 25 mM DTT) before added with 85 *µ*l of 1x cell buffer (containing 200 mM HEPES, pH 7.4, 1% CHAPS, 50 mM DTT, and 20 mM EDTA) and 5 *µ*l of caspase-3 substrate (Acetyl-Asp-Glu-Val-Asp p-nitroanilide (Ac-DEVD-pNA)). The absorbance values of light yellow colour formed solutions were measured at 405 nm using the ELISA reader.

For caspase-8 and cytochrome c activities, Human Caspase-8/FLICE Platinum ELISA assay kit (eBioscience, Austria) and Human Cytochrome c Platinum ELISA assay kit (eBioscience, Austria) were used, respectively. Briefly, the harvested cells were lysed using 200 *µ*l of 1x lysis buffer. In the caspase-8 analysis, a total of 50 *µ*l cell lysate was added in a 96-microwell plate coated with a monoclonal antibody to human caspase-8, before added with 50 *µ*l of detection antibody, 100 *µ*l of diluted anti-rabbit-IgG-HRP, and 100 *µ*l of TMB substrate solution. Meanwhile, for cytochrome c activity, 50 *µ*l of lysed cells were added in a 96-microwell plate coated with a monoclonal antibody to human cytochrome c, before added with 50 *µ*l of biotin-conjugated, 100 *µ*l of diluted streptavidin-HRP, and TMB substrate solution. The stop solution (100 *µ*l) was added into each well before measured at 450 nm using the ELISA reader. All the final results of treated cells were compared with untreated cells (control) and were expressed as fold of increase in caspases or cytochrome c activity.

### 2.9. p53, Bax, and Bcl-2 Expression

Protein expression of p53, Bax, and Bcl-2 was analyzed based on the flow cytometry protocol for staining intracellular molecules as described by the Research and Development (R&D) system (NY) [20] with slight modification. Each cancer cell line at a density of 5 × 10^5^ cells/well was seeded in a 6-well plate and grown for 24 h at 37°C in an incubator with 5% CO_2_. After the incubation, the cell was treated with selected leaf extract at IC_50_ values and incubated again for 24 h, 48 h, and 72 h. The untreated cell was used as a control. Adhering and floating cells were collected and centrifuged at 2000 rpm in 4°C for 5 min. Briefly, the cell pellet was fixed by adding 100 *µ*l of 1% (w/v) freshly prepared paraformaldehyde and incubated at room temperature (25°C ± 2) for 10 min. The cell pellet was then permeabilized in 100 *µ*l of 1% Triton X-100 for 10 min before proceeding to staining with antibodies. For direct staining of p53 antibody, the permeabilized cell was added with 10 *µ*l of 2.5 *µ*g/ml p53 antibody, followed by incubation in dark condition at room temperature (25°C ± 2) for 30 min. The stained cell was then centrifuged (2000 rpm in 4°C for 5 min) and washed again with cold 1x PBS before added with 400 *µ*l of cold 1x PBS before flow cytometer analysis. For indirect staining of Bax and Bcl-2 antibodies, the permeabilized cell was added with 10 *µ*l of 2.5 *µ*g/ml Bax or Bcl-2 antibodies and incubated in dark condition at room temperature (25°C ± 2) for 30 min. Then, 500 *µ*l of cold 1x PBS was added and centrifuged at 2000 rpm in 4°C for 5 min. Each cell pellet was added with 10 *µ*l of IgG polyclonal anti-mouse secondary antibody and incubated in dark condition at room temperature (25°C ± 2) for another 30 min. The stained cell was washed twice with cold 1x PBS, and the centrifugation step was repeated. Finally, the cell pellet was resuspended in 400 *µ*l of cold 1x PBS before flow cytometer analysis.

### 2.10. Data Analysis

All data were analyzed using GraphPad Prism version 5.01 and expressed as means ± standard deviation (S.D.) of five replicate analyses in five independent experiments. One-way analysis of variance (ANOVA) followed by Tukey's multiple range test was carried out to determine the significance between means. The statistically significant level was set at *P* < 0.05.

## 3. Result

### 3.1. Cytotoxicity Effect of Commercial Teas and *E. longifolia* Leaf Extracts

**Figure 1:**
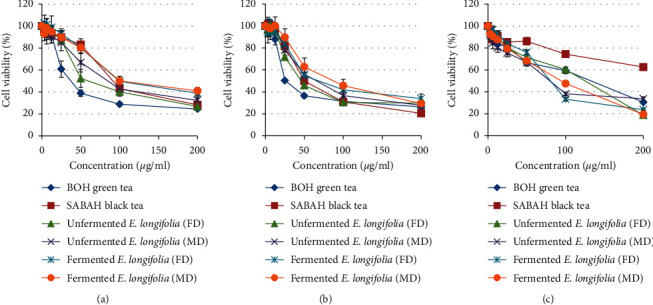
Cell viability after treated with commercial teas and *E. longifolia* leaf extracts against (a) non-hormone-dependent breast cancer cell MDA-MB-231, (b) hormone-dependent breast cancer cell MCF-7, and (c) normal mouse embryonic fibroblast cell NIH-3T3. FD: freeze-dried. MD: microwave-oven dried.

**Figure 2:**
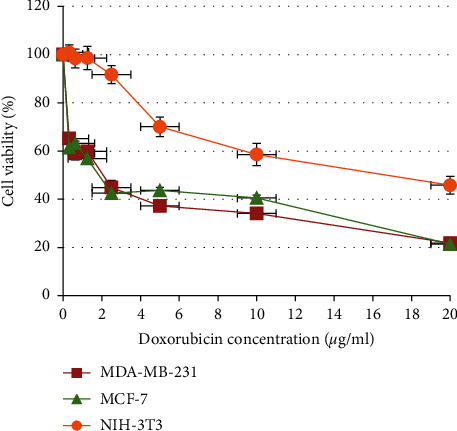
Cell viability after treated with positive control of drug doxorubicin against breast cancer cell lines, MCF-7 and MDA-MB-231, and normal mouse embryonic fibroblast cell NIH-3T3.

In the present study, the percentage of cell viability of MDA-MB-231 (Figure 1(a)), MCF-7 (Figure 1(b)), and NIH-3T3 (Figure 1(c)) displayed a clear decreasing pattern in a dose-dependent manner for all extracts of *C. sinensis* and *E. longifolia* leaves. Doxorubicin at a concentration of 0–20 *µ*g/ml (Figure 2) also displayed a similar pattern with IC_50_ values less than 3 *µ*g/ml for both cancer cell lines.

The most prominent result was shown by the inhibition in the unfermented freeze-dried leaf extract of *E. longifolia* on the cell viability of breast cancer cell line, especially against MCF-7 cells with an IC_50_ value of 45.0 ± 3.5 *µ*g/ml (Table 1). The amount of this extract was lower compared to SABAH black tea (50.5 ± 0.5 *µ*g/ml). Besides, this extract also showed a potent cytotoxicity effect against MDA-MB-231 cells with an IC_50_ value of 69.3 ± 17.2 *µ*g/ml and significantly lower compared to SABAH black tea (92.3 ± 3.2 *µ*g/ml). Both cancer cell lines displayed a similar pattern, where the freeze-dried leaf extract showed lower IC_50_ values compared to those microwave-oven dried regardless it was fermented or not. Still, fermented leaves always possess higher values than those were unfermented.

### 3.2. Phenolic Determination of Commercial Teas and *E. longifolia* Leaf Extracts

In the phenolic determination, complete baseline separation of an individual compound by HPLC-DAD was obtained by a combination of gradient and isocratic elution. The retention time of different compounds were 3.565 min for gallic acid, 4.203 for chlorogenic acid, 5.726 for (-)-epicatechin (EC), 6.066 for (-)-epigallocatechin gallate (EGCG), 6.878 for caffeic acid, 7.190 for vanillic acid, 7.595 for (-)-epigallocatechin (EGC), 10.339 for (-)-epicatechin gallate (ECG), 10.696 for *p*-coumaric acid, 12.041 for ferulic acid, 13.401 for naringin, and 13.818 for hesperidin. Based on Table 2, phenolics in the unfermented freeze-dried leaf extract such as gallic acid (9.81 ± 0.02 mg/L), chlorogenic acid (7.70 ± 0.08 mg/L), and catechin derivatives of ECG (3.07 ± 0.02 mg/L) and EGCG (2.28 ± 0.01 mg/L) were recorded highest compare to other types of *E. longifolia.*

### 3.3. Effect of Unfermented Freeze-Dried Leaf Extract of *E. longifolia* at IC_50_ Values on DNA Fragmentation

**Figure 3:**
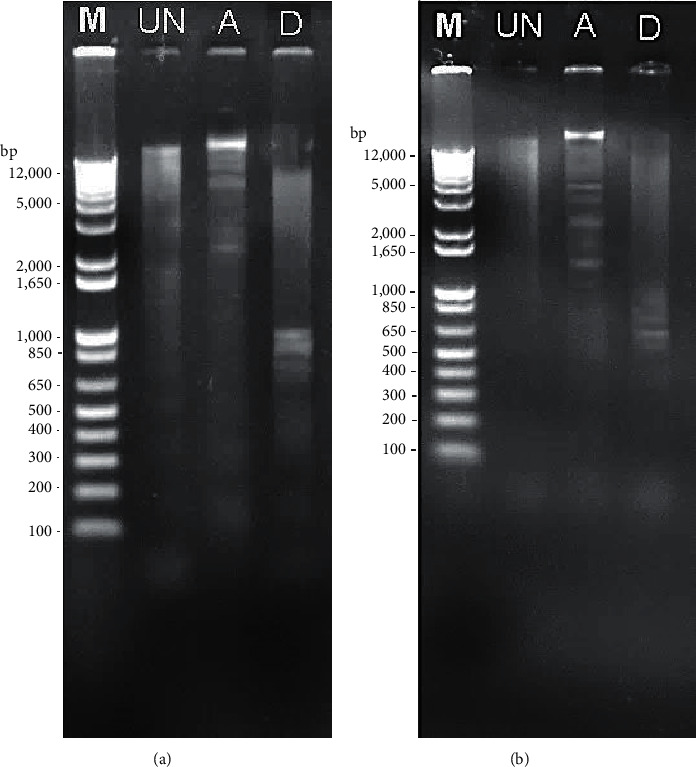
DNA fragmentation of (a) MDA-MB-231 and (b) MCF-7 cell lines after treated with unfermented freeze-dried leaf extract of *E. longifolia* at IC_50_ value for 72 h. M: DNA marker; UN: untreated; A: unfermented freeze-dried leaf extract of *E. longifolia*; D: positive control drug doxorubicin.

In DNA laddering analysis, all treated MDA-MB-231 and MCF-7 cell lines showed DNA fragmentation after 72 h of treatment with the extract. Treated MDA-MB-231 cells developed multiple DNA bands ranging from 2000 bp to >12,000 bp with RNA smear (Figure 3(a)). Similarly, treated MCF-7 cells also developed multiple DNA bands ranging from 1000 bp to >12,000 bp with RNA smear (Figure 3(b)).

### 3.4. Effect of Unfermented Freeze-Dried Leaf Extract of *E. longifolia* at IC_50_ Value on Cell Cycle Distribution and Apoptosis

**Figure 4:**
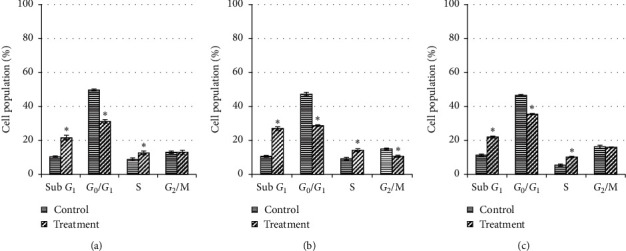
Cell cycle distribution of untreated MDA-MB-231 cells and those treated with unfermented freeze-dried leaf extract of *E. longifolia* at IC_50_ value for (a) 24 h, (b) 48 h, and (c) 72 h. The data are presented as mean ± standard deviation for three replicates and indicated by an asterisk showed a significant difference (*P* < 0.05) relative to respective control.

**Figure 5:**
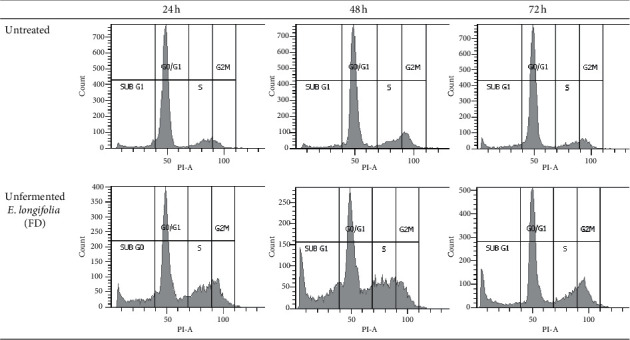
Representative flow cytometric scan of untreated MDA-MB-231 cells and those treated with unfermented freeze-dried leaf extract of *E. longifolia* at IC_50_ value for 24 h, 48 h, and 72 h. FD: freeze-dried.

For MDA-MB-231 cells (Figures 4 and 5), a significant cell cycle arrest at S phase was observed as early as 24 h (12.6%) and slightly increased at 48 h (14.2%) before decrease at 72 h of treatment (10.2%). At the meantime, the number of cells in *G*_0_/*G*_1_ and *G*_2_/M phases was reduced significantly at 48 h compared to 24 h treatment and showed a moderate increment at 72 h. As early as 24 h treatment, the apoptosis (sub-*G*_1_ phase) increases from 21.6% to 27.0% at 48 h but decreases to 22.0% at 72 h; however, all were significantly higher (*P* < 0.05) compared to their respective controls (10.4%, 10.6%, and 11.3%). This indicates that the selected extract at IC_50_ value was more effective to induce apoptosis at 48 h of treatment compared to 24 h or 72 h.

**Figure 6:**
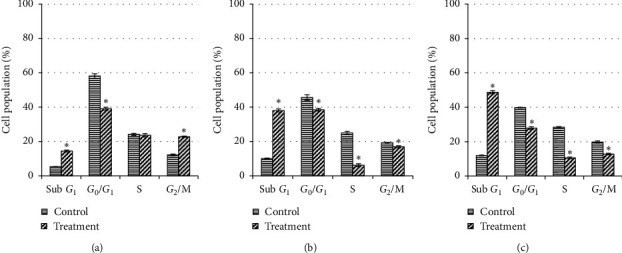
Cell cycle distribution of untreated MCF-7 cells and those treated with unfermented freeze-dried leaf extract of *E. longifolia* at IC_50_ value for (a) 24 h, (b) 48 h, and (c) 72 h. The data are presented as mean ± standard deviation for three replicates and indicated by an asterisk showed a significant difference (*P* < 0.05) relative to respective control.

**Figure 7:**
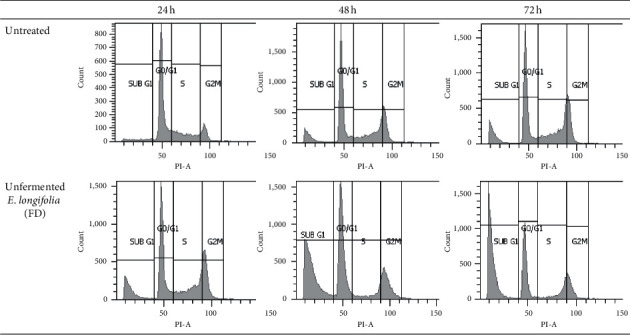
Representative flow cytometric scan of untreated MCF-7 cells and those treated with unfermented freeze-dried leaf extract of *E. longifolia* at IC_50_ value for 24 h, 48 h, and 72 h. FD: freeze-dried.

In contrast, MCF-7 cells (Figures 6 and 7) showed a significant arrest at *G*_2_/M phase at 24 h of treatment (22.8%) (*P* < 0.05) compared to control before suddenly decrease and shifting to apoptosis (sub *G*_1_) with the drastic increase at 48 h (16.9%) and 72 h (12.8%) of treatment. Meanwhile, the numbers of cell in *G*_0_/*G*_1_ and S phases were reduced significantly (*P* < 0.05). Apoptosis also occurred during *G*_2_/M arrest for the first 24 h of the treatment (14.4%) and significantly increased up to 38.0% at 48 h and 48.8% at 72 h compared to their respective controls (5.3%, 10.1%, and 12.0%) (*P* < 0.05). Also, the proportion of cells in *G*_0_/*G*_1_ and *G*_2_/M phases was decreased significantly (*P* < 0.05) compared to their control after 24 h, 48 h, and 72 h of treatment.

### 3.5. Effect of Unfermented Freeze-Dried Leaves of *E. longifolia* at IC_50_ Value on Early and Late Apoptosis

**Figure 8:**
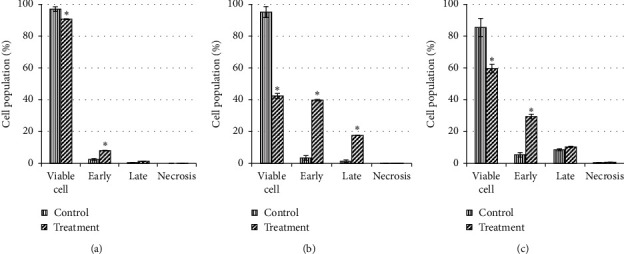
The cell population of untreated MDA-MB-231 cells and those treated with unfermented freeze-dried leaf extract of *E. longifolia* at IC_50_ value for (a) 24 h, (b) 48 h, and (c) 72 h. The values are presented as mean ± standard deviation for three replicates and indicated by an asterisk showed a significant difference (*P* < 0.05) relative to respective control.

**Figure 9:**
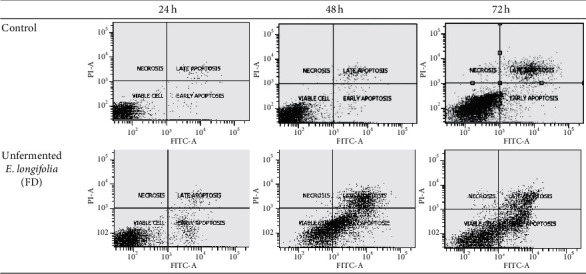
Representative flow cytometric scan of untreated MDA-MB-231 cells and those treated with unfermented freeze-dried leaf extract of *E. longifolia* at IC_50_ value for 24 h, 48 h, and 72 h.

The exposure of unfermented freeze-dried leaf extract of *E. longifolia* on MDA-MB-231 cells (Figures 8 and 9) for 24 h showed significant (*P* < 0.05) small percentage of early apoptosis (8.0%) event occurred when compared to the control (2.5%). Following 48 h of treatment, it has resulted in a substantial shift from live cells to early and late apoptotic cell populations with the value of 39.8% and 17.6%, respectively (*P* < 0.05). However, total apoptosis decreased to a level of 39.8% (early apoptosis: 29.4%; late apoptosis: 10.4%) for 72 h of treatment.

**Figure 10:**
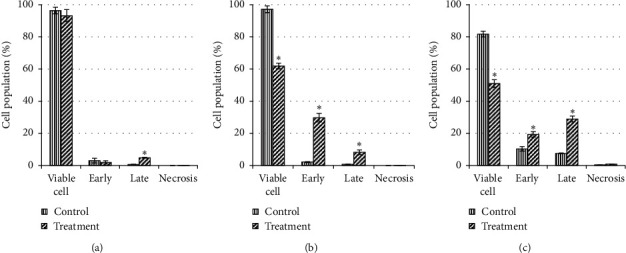
The cell population of untreated MCF-7 cells and those treated with unfermented freeze-dried leaf extract of *E. longifolia* at IC_50_ value for (a) 24 h, (b) 48 h, and (c) 72 h. The values are presented as mean ± standard deviation for three replicates and indicated by an asterisk showed a significant difference (*P* < 0.05) relative to respective control.

**Figure 11:**
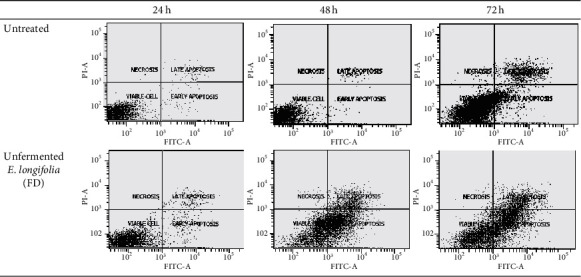
Representative flow cytometric scan of untreated MCF-7 cells and those treated with unfermented freeze-dried leaf extract of *E. longifolia* at IC_50_ value for 24 h, 48 h, and 72 h. FD: freeze-dried.

For MCF-7 cells (Figures 10 and 11), after 24 h exposure of this extract, there was a significant (*P* < 0.05) late apoptosis event (4.8%) occurred when compared to the control (0.6%). Following 48 h of treatment, treated cells showed a strong shift from live cells to early and late apoptotic cell populations with the value of 29.7% and 8.2%, respectively (*P* < 0.05). After 72 h of treatment, the total apoptosis increased up to 48.2% (early apoptosis: 19.3%; late apoptosis: 28.9%). This suggested the apoptosis in MCF-7 cells occurred in a time-depended manner.

### 3.6. Possible Anticancer Mechanism of Unfermented Freeze-Dried Leaf Extract of *E. longifolia* at IC_50_ Value on the Activity of Caspase-3, Caspase-8, and Cytochrome c and Also Protein Expression of Bcl-2, Bax, and p53

**Figure 12:**
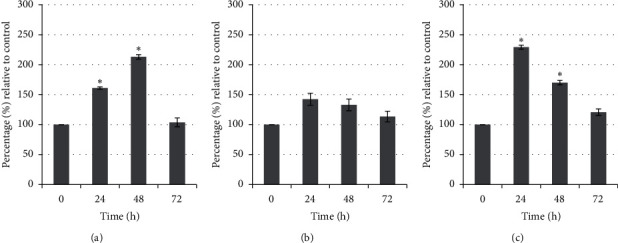
(a) Caspase-3, (b) caspase-8, and (c) cytochrome c activities of MDA-MB-231 cells treated with unfermented freeze-dried leaf extract of *E. longifolia* at IC_50_ value. The values are presented as mean ± standard deviation for three replicates and indicated by an asterisk showed a significant difference (*P* < 0.05) relative to respective control.

**Figure 13:**
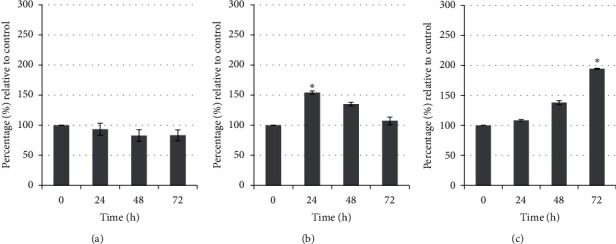
(a) Caspase-3, (b) caspase-8, and (c) cytochrome c activities of MCF-7 cells treated with unfermented freeze-dried leaf extract of *E. longifolia* at IC_50_ value. The values are presented as mean ± standard deviation for three replicates and indicated by an asterisk showed a significant difference (*P* < 0.05) relative to respective control.

Treated MDA-MB-231 cells showed the activation (*P* < 0.05) of caspase-3 at 24 h and increase at 48 h with 2-fold than the control but decrease after 72 h of treatment. In contrast, the cytochrome c activity was higher (*P* < 0.05) at 24 h but decreased at 48 h and 72 h of treatment. Only caspase-8 showed no activity in MDA-MB-231 cells throughout the treatment periods (Figure 12). Differently, treated MCF-7 cells showed activities in caspase-8 and cytochrome c but no activation of caspase-3 throughout the treatment periods. The cytochrome c activity was increased with time and led to more than 2-fold (*P* < 0.05) activity at 72 h of treatment. In contrast, caspase-8 activity was decreased with time compared to their respective controls (Figure 13).

**Figure 14:**
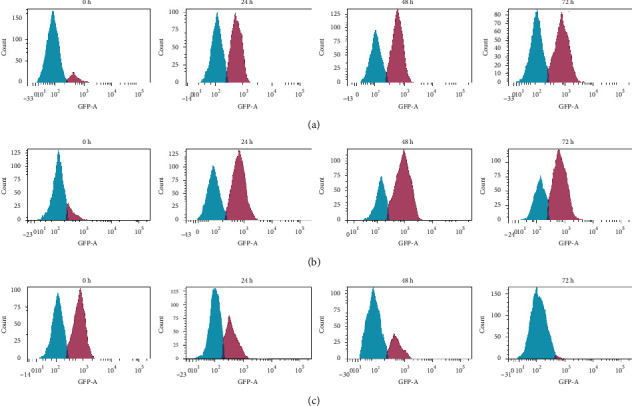
Representative flow cytometric scan of untreated MDA-MB-231 cells and those treated with unfermented freeze-dried leaf extract of *E. longifolia* at IC_50_ value for 24 h, 48 h, and 72 h. (a) p53, (b) Bax, and (c) Bcl-2 expressions. Blue peak = unstained cells. The purple peak at 0 *h* = untreated cells. Purple peaks at 24, 48, and 72 *h* = treated cells with extract at the respective treatment period.

**Figure 15:**
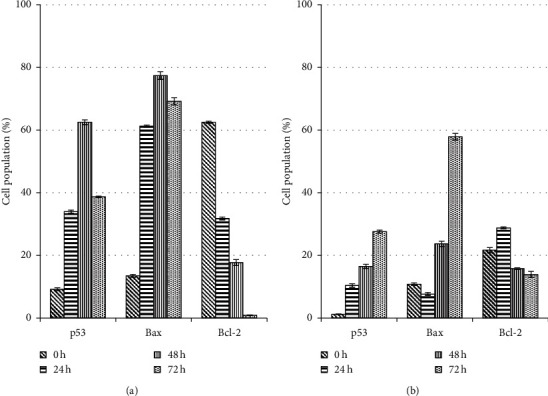
p53, Bax and Bcl-2 proteins expression of A. MDA-MB-231 and B. MCF-7 cancer cell lines treated with unfermented freeze-dried leaf extract of E. longifolia at IC50 value. -e values are presented as mean ± standard deviation for three replicates and indicated by an asterisk were showed a significant difference (P < 0.05) relative to respective control (values at 0 h).

The extract caused the increase of Bax and p53 expression in MDA-MB-231 cells from 24 h (34.0% and 61.3%, respectively) to 48 h (62.5% and 77.4%, respectively) but reduced moderately after 72 h (38.7% and 69.2%, respectively) (Figures 14 and 15(a)). Meanwhile, the expression of Bcl-2 was gradually decreased through the treatment periods of 24 h, 48 h, and 72 h (31.8%, 17.7%, and 0.9%, respectively) (*P* > 0.05).

**Figure 16:**
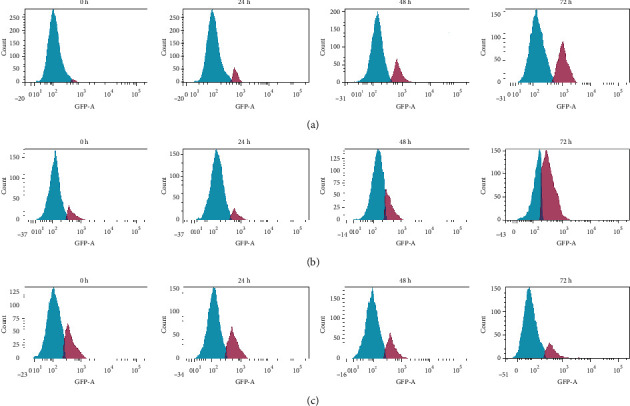
Representative flow cytometric scan of untreated MCF-7 cells and those treated with unfermented freeze-dried leaf extract of E. longifolia at IC_50_ value for 24 h, 48 h and 72 h. (a) p53, (b) Bax and (c) Bcl-2 expressions. Blue peak = unstained cells. The purple peak at 0 h = Untreated cells. The purple peak at 24, 48 and 72 h = Treated cells with extract at the respective treatment period.

Treated MCF-7 showed the expression of Bcl-2 in an antagonistic manner with the p53 and Bax, with decreases of Bcl-2 and increases of p53 and Bax activities throughout the treatment periods of 24 h, 48 h, and 72 h, which significantly different to respective controls (*P* < 0.05) (Figures 16 and 15(b)). In contrast with treated MDA-MB-231 cells, the caspase-3 was not activated in the treated MCF-7 cells. There was the only activation of caspase-8 and cytochrome c.

## 4. Discussion

As the result of cytotoxicity effect evaluation, the leaf extract of *E. longifolia,* which was unfermented and freeze-dried had been further studied for its possible anticancer mechanism of action. The selection was made as it is fulfilling three crucial criteria: (1) possessed the lowest IC_50_ value than the other extracts which indicate the most substantial cytotoxic effect, (2) IC_50_ value was lower than 100 *µ*g/ml of sample concentration (50% less than total concentration tested of 200 *µ*g/ml), and (3) IC_50_ value was lower than IC_50_ value of NIH-3T3 cell (125.5 ± 6.2 *µ*g/ml) indicated that this extract at IC_50_ value was not toxic to normal cell line. The phenolics such as gallic acid, chlorogenic acid, vanillic acid, and catechin derivatives (i.e., ECG and EGCG) that were determined in this extract might contribute to the cytotoxicity effect against MCF-7 and MDA-MB-231 cancer cell lines. Previously, many of these compounds were proven scientifically to inhibit the growth of breast cancer cells [21, 22]. A study by Rezaei-Seresht et al. [23] had shown the cytotoxicity effect of caffeic acid and gallic acid against MCF-7 cells by the activation of intrinsic apoptotic signalling pathways along with the expression of p53, Mcl-1, and p21 genes. Similarly, a study by Schröder et al. [24] also had reported the cytotoxicity effect of ECGC derived from green tea on both MCF-7 and MDA-MB-231 cancer cells, which might be caused by the activation of estrogen receptor-independent pathways.

Apart from phenolics determined in the present study, a recent study by Supartini et al. [25] had revealed the presence of flavonoids, tannins, triterpenoid, carotenoid, kumarin, carbohydrate, and saponin in both ethanolic and water extracts of *E. longifolia* leaves. Moreover, 3 major chemical compounds in its ethanol extract were identified, i.e., 4H-pyran-4-one, 2,3-dihydro-3,5-dihydroxy-6-methyl- (6.78%), 2-cyclohexene-1-one, 4-hydroxy-3,5,6-trimethyl-4-(3-oxo-1-butenyl)- (6.46%), and acetic acid, 2-(2,2,6-trimethyl-7-oxabicyclo[4.1.0]hept-1-yl)-propenyl ester (5.61%). They stated that these compounds had been numerously reported to have biological activities such as antimicrobial, anti-inflammatory, strong antioxidant capacity, anticancer, antimutagenic, antipeptic, antiseptic, antispasmodic, antiandrogenic, and hypocholesterolemic activities. In a different study by Jiwajinda et al. [26], the ethanolic extract of *E. longifolia* leaves consisted of 7 quassinoid compounds of lonilactone, 6-dehydro lonilactone, 11-dehydroklaineanone, 12-epi dehydroklaineanone, 15*β*-hydroxyklaineanone, 14,15 *β*-dihydroxyklaineanone, and 15-*β*-O-acetyl-14-hydroxyklaineanone. Quassinoids are a well-known compound to exhibit a wide range of inhibitory effects, including anti-inflammatory, antiviral, antimalarial, and antiproliferative effects on various cancer cell types including breast cancer [27]. Meanwhile, Zakaria et al. [28] have detected the presence of eurycomanone and 14,15*ß*-dihydroxyklaineanone in leaf extracts of *E. longifolia*. These compounds have also been found significantly in its roots, where eurycomanone, in particular, had been proven to exert antiproliferation or cytotoxicity effect against breast carcinoma cell of MCF-7 [29].

However, those phenolics appear to be affected by processing factors since the present result of the cytotoxicity effect between different types of *E. longifolia* extracts was obvious. The steam blanching process acts as the pretreatment to deactivate the degradative enzymes called polyphenol oxidase (PPO) to prevent the oxidation of polyphenol compounds and retaining more phenolic content in the unfermented leaves. Differently, the absence of this crucial step in the fermented leaf preparation caused the deterioration of phenolic compounds. The withering and fermentation processes also might cause the oxidation or degradation of phenolic compounds in fermented leaves [30]. In terms of drying techniques, the nonthermal of freeze-drying gave the end-product with higher phenolic content compared to the microwave-oven drying due to its low temperature, vacuum conditions and very low deterioration reaction rates [31]. The primary structure of dried leaves was preserved during drying due to the developed frozen water molecules or ice crystal within the leaf tissue matrix has lower movement compared to its liquid form [31]. This present finding was in agreement with a recent study by Roslan et al. [32], as the freeze-dried leaves of *Camellia sinensis* or authentic tea showed the highest phenolics, flavonoids, and antioxidant activity compared to superheated steam and oven drying techniques.

At the cellular level, chemotherapeutic agents act primarily by inducing cancer cell death through the mechanism of apoptosis [33]. In the present study, there was an evident selected *E. longifolia* extract was able to induce apoptosis in both breast cancer cells: MCF-7 and MDA-MB-231. DNA fragmentation or the cleavage of DNA into oligonucleosomal size fragments suggesting this extract had caused the cells to undergo apoptosis cell death mode. The dying cells during apoptosis would leave a minimum impact to neighbouring tissues *in vivo* compared to those of necrosis, another form of cell death, which accompanied by membrane rupture and leakage of cellular contents causing tissue inflammation [34], thus making it a promising agent for chemotherapy, which merits further study.

A flow cytometry result showed the number of apoptotic cells by calculation based on the appearance of cells in *G*_1_. The number of apoptotic MDA-MB-231 cells was increased as the treatment period was prolonged from 24 h to 48 h but decreased at 72 h, which also displayed in the similar manner of S phase cells in population, indicated that the S phase was arrested as the synthesis of DNA was inhibited and the cells were prevented from entering *G*_2_/M phase [35, 36]. The activity of Bax increased from 24 h to 48 h but decreased of Bcl-2 at the same time suggesting that this extract induced the upregulation of Bax and downregulation of Bcl-2. The Bax gene is a transcriptional target of p53 and could be upregulated in response to p53-dependent apoptosis triggers. Meanwhile, the downregulation of Bcl-2 has caused the release of cytochrome c from mitochondria into the cytosol. In turn, the release of cytochrome c binds with Apaf-1 which activates caspase-9 before activates downstream effector caspases such as caspase-3, -6, and -7 [37]. In this current study, the activated caspase-3 might then lead to apoptotic cell death. A similar finding in Birjandian et al.'s research [38] had shown the crude methanolic extract of *Echinophora platyloba* promotes MDA-MB-231 cell death at IC_50_ 534.6 ± 7.2 *µ*g/mL by inducing cell arrest at S phase after 24 h of incubation and significantly upregulates the expression of Bax and p27 gene, as well as the downregulation of Bcl-2 gene expression.

Differently, the percent of MCF-7 apoptotic cells rose as the time of treatment was prolonged from 24 h, 48 h, and then 72 h, similarly with the distribution of *G*_2_/M phase cells in its population, being evident that the *G*_2_/M phase became the dominant phase during apoptosis in treated cells in a time-dependent manner. These findings indicate that the cytotoxicity effect of this extract on MCF-7 cells could be attributed primarily to the induction of *G*_2_/M arrest. MCF-7 cells got accumulated at *G*_2_/M phase as the DNA was damaged or unreplicated and prevented cells from undergoing mitosis process [18, 39]. In contrast with the treated MDA-MB-231 cells, the caspase-3 was not activated in treated MCF-7 cells. There was the only activation of caspase-8 and cytochrome c. There is a possibility that this extract might induce two apoptosis pathways, i.e., caspase-8-initiated pathway and mitochondrial-initiated caspase-9-mediated pathway [40, 41]. Moreover, since the caspase-3 was not activated, another downstream effector caspase-6 and -7 might be triggering the apoptosis initiation [37]. Ali et al. [42] have also reported that a curcumin derivative of (*Z*)-3-hydroxy-1-(2-hydroxyphenyl)-3-phenylprop-2-en-1-one (DK1) activates the upregulation of p53 and p21 and downregulation of PLK-1 which promote phosphorylation of CDC2 which caused the arrest of *G*_2_/M phase. Besides, results of increase reactive oxygen species and decrement of antioxidant glutathione level were associated with apoptosis along with elevation of cytochrome c and then activation of caspase-9.

## 5. Conclusion

The unfermented freeze-dried leaf extract of *E. longifolia* was determined as the most effective anticancer source against MDA-MB-231 and MCF-7 cell lines compared to other *E. longifolia* extracts. The extract was proven to induce apoptosis cell death which leads to DNA fragmentation. A significant cell cycle arrest at S phase was observed as early as 24 h for MDA-MB-231 which was caused by the failure of DNA synthesis. In contrast, the cell cycle arrest on *G*_2_/M of MCF-7 indicates that DNA was damaged and unable to be duplicated. The upregulation of Bax and downregulation of Bcl-2 expression in MDA-MB-231 cells might be responsible for cytochrome c and caspase-3 activities which lead to apoptosis cell death. The actions of caspase-8 and cytochrome c in MCF-7 with upregulation of Bax and p53 and also downregulation of Bcl-2 expression showed the possibility of this extract might induce two apoptosis pathways, i.e., caspase-8-initiated pathway and mitochondrial-initiated caspase-9-mediated pathway, triggering downstream effector caspase-6 and -7 and initiating the apoptosis mechanism. These results indicate the ability of unfermented freeze-dried leaf extract of *E. longifolia* to induce apoptosis cell death on MDA-MB-231 and MCF-7, as well as providing fundamental evidence on the sample preparation effect towards its cytotoxicity level. In future, these data can act as initial guidance towards developing chemotherapeutic agent for cancer treatment. Additional *in vivo* studies on the toxicology, bioavailability, and anticancer activity are however needed to clarify the efficacy and safety of this extract in breast cancer treatment.

## Figures and Tables

**Table 1:** IC_50_ (*µ*g/ml) of commercial teas and *E. longifolia* leaf extracts against breast cancer cell lines, MCF-7 and MDA-MB-231, and normal mouse embryonic fibroblast cell NIH-3T3.

Sample	MDA-MB-231	MCF-7	NIH-3T3
Doxorubicin	2.1 ± 0.07^f^	1.8 ± 0.01^e^	15.4 ± 1.45^e^

Commercial tea (*C. sinensis*)			
BOH green tea	35.0 ± 5.3^e^	26.2 ± 2.0^d^	133.3 ± 5.6^a^
SABAH black tea	92.3 ± 3.2^bc^	50.5 ± 0.5^bc^	>200

*E. longifolia*			
Unfermented (FD)	69.3 ± 17.2^d^	45.0 ± 3.5^c^	125.5 ± 6.2^b^
Unfermented (MD)	84.7 ± 8.4^c^	65.7 ± 20.3^b^	79.7 ± 7.2^d^
Fermented (FD)	98.3 ± 11.5^ab^	66.7 ± 20.5^b^	81.8 ± 16.2^cd^
Fermented (MD)	99.7 ± 6.8^a^	97.3 ± 13.6^a^	96.4 ± 3.6^c^

Values are expressed as mean ± standard deviation (*n* = 5); means with different superscript lowercase letters within the same column were significantly different at the level of *P* < 0.05. FD: freeze-dried. MD: microwave-oven dried.

**Table 2:** Phenolic content (mg/L) in commercial teas and *E. longifolia* leaf infusions.

Sample	Commercial tea (*Camellia sinensis*)		*Eurycoma longifolia*			
	BOH green tea	SABAH black tea	Unfermented (FD)	Unfermented (MD)	Fermented (FD)	Fermented (MD)
Gallic acid	18.50 ± 0.07^b^	19.23 ± 0.05^a^	9.81 ± 0.02^c^	8.53 ± 0.06^d^	9.78 ± 0.02^c^	7.52 ± 0.02^e^
Chlorogenic acid	ND	ND	7.70 ± 0.08^b^	4.36 ± 0.03^d^	8.00 ± 0.09^a^	4.98 ± 0.09^c^
*p*-Coumaric acid	9.43 ± 0.01^a^	5.71 ± 0.01^b^	ND	ND	ND	ND
Vanillic acid	ND	ND	5.44 ± 0.01^a^	5.51 ± 0.01^a^	5.23 ± 0.01^b^	5.29 ± 0.01^b^
Caffeic acid	ND	ND	ND	ND	ND	ND
Ferulic acid	ND	ND	ND	ND	ND	ND
EC	88.95 ± 0.15^a^	83.13 ± 0.11^b^	ND	ND	ND	ND
ECG	23.60 ± 0.16^a^	12.51 ± 0.14^b^	3.07 ± 0.02^c^	2.66 ± 0.03^d^	ND	ND
EGC	173.36 ± 1.13^b^	240.01 ± 0.77^a^	ND	ND	ND	ND
EGCG	314.89 ± 0.88^a^	160.74 ± 0.57^b^	2.28 ± 0.01^c^	1.55 ± 0.01^d^	ND	ND
Hesperidin	ND	ND	ND	ND	ND	ND
Naringin	ND	ND	ND	ND	ND	ND

EC: (-)-epicatechin; EGC: (-)-epigallocatechin; ECG: (-)-epicatechin gallate; EGCG: (-)-epigallocatechin gallate; FD: freeze-dried; MD: microwave-oven dried; ND: not detected; values are expressed as mean ± standard deviation (*n* = 5); means with different superscript lowercase letters within the same row were significantly different at the level of *P* < 0.05.

## Data Availability

The datasets generated and analyzed during the current study are available from the first author on reasonable request.
